# Prevalence and epidemiology of hepatitis C in Uzbekistan: results from a population-based serosurvey, 2022

**DOI:** 10.1016/j.ijregi.2026.100968

**Published:** 2026-07-30

**Authors:** Shaun Shadaker, Elizaveta Joldasova, Rania A. Tohme, Aybek Khodiev, Shakhida Karamatova, Nataliya Kan, Abdulaziz Abdurasulov, Botirjon Kurbanov, Zulfiya Abdurakhimova, Dilafkor Mirdjalilov, Saleem Kamili, Nino Khetsuriani, Erkin Musabaev

**Affiliations:** 1 Division of Viral Hepatitis, Centers for Disease Control and Prevention, Atlanta, Georgia; 2 Research Institute of Virology of the Republican Specialized Scientific and Practical Medical Centre of Epidemiology, Microbiology, Infectious and Parasitic Diseases, Tashkent, Uzbekistan; 3 Integral Global Health, Tashkent, Uzbekistan; 4 Division of Global Health Protection, Centers for Disease Control and Prevention, Uzbekistan Country Office, Tashkent, Uzbekistan; 5 Committee for Sanitary and Epidemiological Welfare and Public Health, Ministry of Health of the Republic of Uzbekistan, Tashkent, Uzbekistan; 6 Tashkent Medical Academy, Department of Public Health and Management, Tashkent, Uzbekistan

**Keywords:** Hepatitis C, Prevalence, Epidemiology, Serosurvey, Uzbekistan

## Abstract

**Objectives:**

Uzbekistan launched a national viral hepatitis elimination program in 2022, but precise estimates of hepatitis C prevalence are needed to guide elimination efforts. In 2022, we conducted a cross-sectional population-based seroprevalence survey among adults (aged ≥18 years) and children (aged 5-17 years) in Uzbekistan to estimate the prevalence and epidemiology of hepatitis C.

**Methods:**

Interviewers collected information on demographics and potential exposures to hepatitis C virus (HCV). Blood samples were tested for HCV antibodies (anti-HCV) and, if positive, for HCV RNA. We calculated weighted prevalence estimates and 95% confidence intervals (CIs) and assessed factors associated with HCV exposure and infection using multivariable logistic regression.

**Results:**

Overall, 9066 adults and 4087 children were tested for hepatitis C. The prevalence of anti-HCV and HCV RNA among adults was 3.9% (95% CI: 3.3-4.6) and 2.0% (95% CI: 1.6-2.6), respectively, and among children was 1.1% (95% CI: 0.5-2.2) and 0.5% (95% CI: 0.2-1.0), respectively. Prevalence of anti-HCV and HCV RNA increased with age. Ever having surgery or an invasive medical procedure (adjusted odds ratio [aOR] 1.65 [95% CI: 1.09-2.50]), and past incarceration (aOR 4.88 [95% CI: 1.46-16.29]) were associated with HCV exposure, whereas a history of invasive dental procedures (aOR 2.63 [95% CI: 1.11-6.22]) and receipt of a tattoo (aOR 3.84 [95% CI: 1.57-9.38]) were associated with chronic infection.

**Conclusion:**

Uzbekistan could consider nationwide HCV screening, focusing first on older age groups and incarcerated persons, and implementing infection prevention and control measures in health care settings to help prevent future infections.

## Highlights

• Anti-hepatitis C virus (HCV) prevalence in Uzbekistan in 2022 was 3.9% for adults and 1.1% for children.
• HCV ribonucleic acid prevalence in Uzbekistan in 2022 was 2.0% for adults and 0.5% for children.
• Past surgery, invasive medical procedure, or incarceration was associated with HCV exposure.
• Invasive dental procedures and receipt of a tattoo were associated with HCV infection.

## Graphical Abstract

**Figure:**
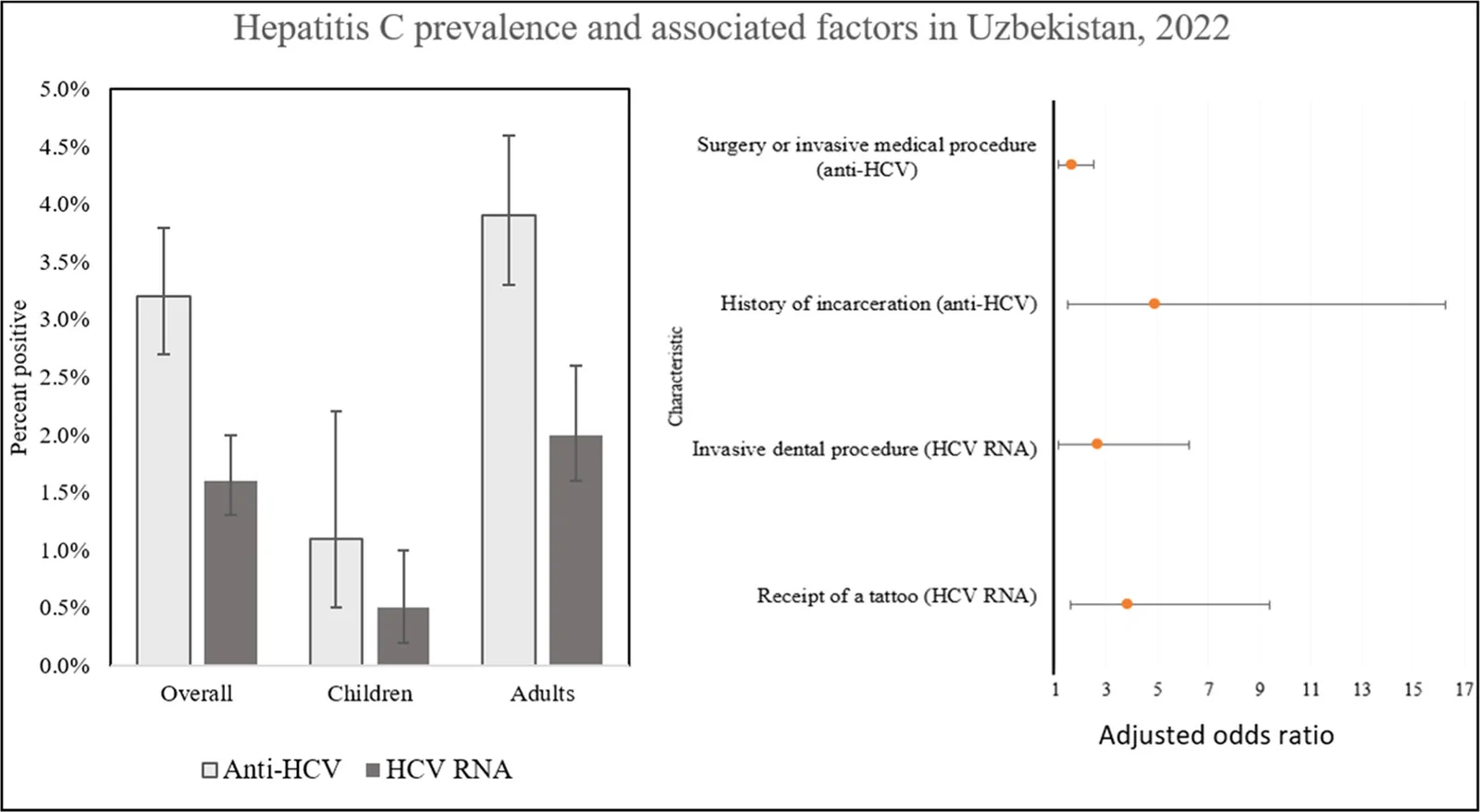

## Introduction

Infection with hepatitis C virus (HCV) is a major global health problem. In 2019, an estimated 58 million people globally were living with HCV infection, and 290,000 people died from infection-related causes, including cirrhosis and liver cancer [1]. To combat this problem, a global target has been set to eliminate hepatitis C as a public health problem by 2030 [2], including an 80% reduction in HCV incidence and a 65% reduction in mortality [3].

Uzbekistan, a country in Central Asia with a population of 37.5 million [4], began its work combating the burden of chronic hepatitis B virus and HCV infections in 2017 through a decree that aimed to improve prevention, diagnostic, and treatment measures during 2017-2021 [5]. As part of those efforts, in December 2019, a limited one-year pilot program for viral hepatitis testing and treatment was initiated in the capital of Tashkent, which showed the feasibility of an elimination program based on rapid tests and a simplified protocol if treatment costs were affordable for patients [6,7]. Following the expansion of this program to six other regions, in 2022, a presidential decree launched a national program for viral hepatitis elimination [7,8]. To bolster elimination efforts, since 2023, the general population of Uzbekistan has been able to receive free screening and diagnostic testing for viral hepatitis, as well as free treatment for hepatitis C through the national elimination program [9]. To guide these efforts, robust prevalence estimates of hepatitis C in Uzbekistan are needed. A systematic review conducted in 2019 estimated the prevalence of HCV antibodies (anti-HCV) among the general population to be 9.6%, with previous estimates ranging anywhere from 6.4% to 13.1% [10]. However, the systematic review used data from studies conducted during 1999-2000, and none of these estimates were from population-based studies. The aforementioned 2019-2020 pilot project suggested that the prevalence of anti-HCV in the capital city of Tashkent was 3% [7]. More recent estimates rely largely on modeled data, which have estimated a national HCV RNA prevalence of 1.1% and approximately 373,000 persons living with HCV infection in Uzbekistan in 2022 [11]. Robust national estimates among the general population are lacking.

A better understanding of the burden and characteristics of hepatitis C in the country will allow for the development of the best approaches for targeted testing and treatment interventions to optimize program results. A more robust assessment of the number and proportion of persons needing HCV treatment in Uzbekistan is also essential for determining the scope and needs of its national hepatitis C elimination program. We conducted a survey to estimate the prevalence and describe the epidemiology of hepatitis C to guide national elimination efforts in Uzbekistan.

## Materials and methods

### *Sample selection*

We conducted a two-stage, cross-sectional seroprevalence survey for coronavirus disease 2019, hepatitis B, and hepatitis C in Uzbekistan in 2022. Seven of the country’s 14 regions were selected based on their expected prevalence of coronavirus disease 2019 and population data to ensure that ≥50% of the population resided in the selected regions. These included Andijan, Samarkand, Kashkadarya, Tashkent, and Khorezm regions, the Republic of Karakalpakstan, and Tashkent city. A sample size of 15,000 was needed to produce estimates for the aggregated seven regions based on an estimated prevalence of anti-severe acute respiratory syndrome coronavirus 2 of 25%, a precision of 1% for a 95% confidence interval (CI), a design effect of 2, and an anticipated 95% response rate. The calculated sample size was confirmed to be sufficient to produce reliable estimates for the hepatitis B and hepatitis C components of the survey; to ensure a sufficient sample size for children, one-third of the total sample (n = 5000) was allocated to children. In the first stage, family polyclinics—institutions in which the entire population within their catchment areas is registered for the provision of primary health care —served as clusters. Overall, 100 polyclinics were selected randomly, with the sample distributed across the seven regions proportionally to their population size and allocated to urban and rural settings according to each region’s population distribution. In the second stage, 150 persons (100 adults and 50 children) were randomly selected from an electronic list of the population served by each polyclinic, where available, or from a patient card index. The selection of respondents was carried out at family polyclinics by the field teams, under the supervision of regional study coordinators.

Individuals aged ≥5 years registered at selected polyclinics were eligible for participation. Those who provided either written or verbal voluntary informed consent to a member of the field team were enrolled. For children, consent was obtained from each child’s parent/legal guardian, paired with verbal assent from children aged 8-17 years. Persons who were unable to give informed consent and those unable to provide a blood sample were excluded.

Data collection took place during March-July 2022. Interviews were conducted face-to-face by trained field personnel using a structured paper-based questionnaire. All participants were asked about their demographic information and medical history; adult participants were also asked about their lifetime behavioral history, including getting tattoos or piercings, injection drug use, and a history of incarceration. Blood samples were collected and linked to questionnaire data using a unique identification number assigned at the time of interview to ensure confidentiality.

### *Laboratory methods*

Venous blood was drawn from participants into labeled serum separator tubes and transported to the laboratory of the Research Institute of Virology in Tashkent. All serum samples were tested for anti-HCV by the Best anti-HCV test kit (AO Vector-Best, Novosibirsk, Russian Federation). Anti-HCV positive samples were tested for HCV RNA by the AmpliSens HCV-FRT Polymerase Chain Reaction (AmpliSens, Moscow, Russian Federation). For quality assurance purposes, specimens that were anti-HCV reactive by Vector-Best were retested for anti-HCV by the ARCHITECT anti-HCV chemiluminescent microparticle immunoassay (Abbott Laboratories, USA), and specimens that had detectable HCV RNA by AmpliSens were tested for HCV core antigen using the ARCHITECT HCV Ag assay (Abbott Laboratories, USA). When a discrepancy between tests was noted, we used the confirmatory test results as the final results. Test results were reported to participants as soon as they were available, and those with current HCV infection were provided post-test counseling and referred to a local health care provider for linkage to care and treatment.

### *Statistical analysis*

Data were weighted to account for the study design. Two-stage sampling weights were created to account for the approach used to sample polyclinics and individuals. Sampling weights were adjusted for non-response. Further, post-stratification weights were used to adjust the sampling weights for noncoverage to ensure that the survey participants appropriately reflected the distribution of the population of the selected seven regions. Direct calibration was performed based on the population joint distribution within each stratum determined by region, sex, and age group for 2022 [4].

Descriptive statistics are presented as weighted proportions and 95% CIs. The chi-square test with an alpha of 0.05 was used to determine significant differences in categorical variables. In the multivariable analysis, all variables associated with anti-HCV or HCV RNA positivity in bivariate analysis were included in the final models to assess factors independently associated with HCV exposure and infection, with adjusted odds ratios (aORs) and 95% CIs. All analyses was performed using SAS version 9.4 (Cary, North Carolina, USA).

This study was approved by the ethics committee of the Ministry of Health of the Republic of Uzbekistan. A review by the Centers for Disease Control and Prevention determined the survey to be public health surveillance and therefore did not constitute human subjects research.

## Results

### *Study population*

Of 15,000 selected participants, 13,153 (87.7%) participated in the survey and were tested for hepatitis C, including 9066 adults (90.7% response rate) and 4087 children aged 5-17 years (81.7% response rate). Demographic characteristics of survey participants as well as weighted population estimates are presented in Table 1. Among adult participants, the median age was 41 years (interquartile range 31-54 years), and 64.2% (n = 5821) were female. The majority were of Uzbek ethnicity (85.2%; n = 7091), 80.6% (n = 6503) were married or living with a partner, and 54.9% (n = 4306) had a university or higher education; 15.8% (n = 1275) were unemployed at the time of the survey. Among children, the median age was 10 years (interquartile range: 7-14), and 51.0% (n = 2086) were female.

**Table 1:** Socio-demographic characteristics of participants in the viral hepatitis serosurvey, Uzbekistan, 2022.

Characteristics	Adults		Children	
	n (%)	Weighted % (95% CI)	n (%)	Weighted % (95% CI)
**Overall**	9066	–	4087	–
**Age, years**				
5-9	–	–	1726 (42.2)	41.6 (37.9-45.5)
10-14	–	–	1599 (39.1)	39.6 (35.0-44.4)
15-17	–	–	762 (18.6)	18.8 (16.5-21.3)
18-29	1898 (20.9)	29.1 (26.8-31.5)	–	–
30-39	2358 (26.0)	25.3 (23.6-27.2)	–	–
40-49	1775 (19.6)	18.0 (16.7-19.4)	–	–
50-59	1542 (17.0)	13.8 (12.4-15.4)	–	–
≥60	1493 (16.5)	13.8 (12.7-14.9)	–	–
**Sex**				
Male	3245 (35.8)	49.4 (46.1-52.7)	2001 (49.0)	51.8 (47.1-56.5)
Female	5821 (64.2)	50.6 (47.3-53.9)	2086 (51.0)	48.2 (43.5-52.9)
**Region**				
Andijan	1555 (17.2)	15.8 (8.7-27.1)	659 (16.1)	15.8 (9.2-25.9)
Kashkadarya	1463 (16.1)	16.1 (10.9-23.2)	580 (14.2)	17.0 (11.1-25.2)
Khorezm	844 (9.3)	9.5 (5.9-14.7)	394 (9.6)	9.8 (6.0-15.4)
Karakalpakstan	911 (10.0)	9.6 (5.2-17.1)	478 (11.7)	10.2 (5.1-19.3)
Samarkand	1546 (17.1)	19.2 (11.8-29.6)	689 (16.9)	20.7 (11.7-34.0)
Tashkent city	1279 (14.1)	14.9 (11.5-19.2)	558 (13.7)	12.6 (9.8-16.0)
Tashkent region	1468 (16.2)	14.8 (10.3-21.0)	729 (17.8)	14.0 (9.5-20.0)
**Ethnicity (N = 8322 adults, 3268 children)**				
Uzbek	7091 (85.2)	87.3 (81.3-91.6)	2,853 (87.3)	85.1 (73.1-92.3)
Other	1231 (14.8)	12.7 (8.4-18.7)	415 (12.7)	14.9 (7.7-26.9)
**Marital status (N = 8070)**				
Never married	866 (10.7)	15.4 (13.6-17.3)	–	–
Married/living with partner	6503 (80.6)	77.1 (75.2-79.0)	–	–
Separated/divorced/widowed	701 (8.7)	7.5 (5.7-9.8)	–	–
**Highest level of education completed (N = 7844)**				
Secondary school or less	218 (2.8)	2.7 (1.9-3.9)	–	–
Professional/technical school	3320 (42.3)	42.8 (36.0-49.8)	–	–
University/college or higher	4306 (54.9)	54.5 (47.4-61.4)	–	–
**Employment status (N = 8054)**				
Employed	3289 (40.8)	43.0 (38.3-47.7)	–	–
Retired	1345 (16.7)	13.2 (11.9-14.6)	–	–
Homemaker	2145 (26.6)	21.4 (18.6-24.4)	–	–
Unemployed/student	1275 (15.8)	22.5 (18.4-27.1)	–	–
**Monthly household income**^a^**(N = 7188)**				
<2,000,000 Som (<178 USD)	2784 (38.7)	43.4 (36.1-51.0)	–	–
2,000,000-4,000,000 Som (178-356 USD)	2002 (27.9)	26.7 (21.0-33.2)	–	–
>4,000,000 Som (>356 USD)	799 (11.1)	9.8 (7.3-13.0)	–	–
Unknown	1603 (22.3)	20.1 (15.1-26.3)	–	–

Abbreviations: CI, confidence interval; USD, US dollars.

a Conversion rate to USD as of May 1, 2022 from https://www.oanda.com/currency-converter.

### *Prevalence of antibody against HCV*

**Figure 1:**
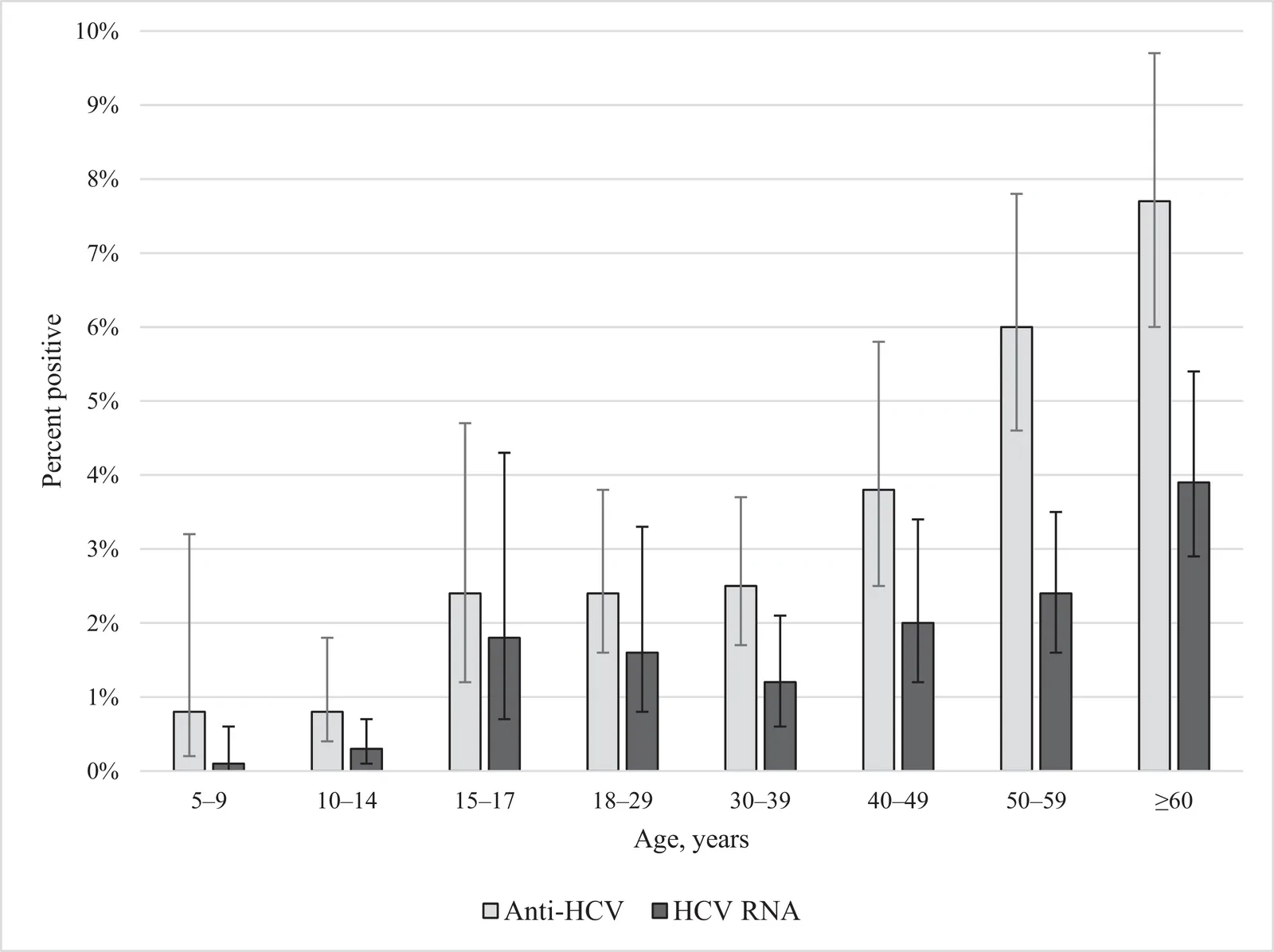
Prevalence of anti-HCV and HCV RNA by age group, Uzbekistan, 2022. Abbreviations: HCV, hepatitis C virus.

Among persons aged ≥5 years, the prevalence of anti-HCV was 3.2% (95% CI: 2.7-3.8). Among adults (≥18 years), the overall prevalence of anti-HCV was 3.9% (95% CI: 3.3-4.6) (Table 2). Anti-HCV prevalence increased with age, from 2.4% (95% CI: 1.6-3.8) among persons aged 18-29 years to 7.7% (95% CI: 6.0-9.7) among those aged ≥60 years (*P* < 0.001) (Figure 1). Anti-HCV prevalence was not significantly different between males (3.6% [95% CI: 2.6-4.9]) and females (4.2% [95% CI: 3.4-5.3]) (*P* = 0.46). Prevalence of anti-HCV varied by region, with the highest prevalence in Khorezm (8.8% [95% CI: 6.7-11.4]) and the lowest in Tashkent region (1.8% [95% CI: 1.1-3.1]) (*P* < 0.001). There were no differences in anti-HCV prevalence by ethnicity or education level. Prevalence of anti-HCV was highest among persons who were married or living with a partner (4.2% [95% CI: 3.4-5.1]) and those who were separated/divorced or widowed (5.3% [95% CI: 3.4-8.1]), as well as those who were retired (7.0% [95% CI: 5.2-9.2]). Persons who had undergone invasive dental or medical procedures, had been previously incarcerated, or had a tattoo had significantly higher prevalence than their counterparts (Table 2). Among children (5-17 years), the overall prevalence of anti-HCV was 1.1% (95% CI: 0.5-2.2) and was highest among those aged 15-17 years (2.4% [95% CI: 1.2-4.7]) (*P*=0.08) (Table 3). Anti-HCV prevalence was not significantly different between male (0.7% [95% CI: 0.4-1.3]), and female (1.5% [95% CI: 0.6-4.0]) children (*P* = 0.15). Prevalence of anti-HCV among children varied by region, with the highest prevalence in Kashkadarya (3.2% [95% CI: 0.9-10.8]).

**Table 2:** Weighted prevalence (% and 95% CI) of hepatitis C among adults by socio-demographic characteristics and practices or exposures that might increase their risk of exposure to hepatitis C, Uzbekistan, 2022.

Characteristics	Total	Anti-HCV+			HCV RNA+		
	N	n	Weighted % (95% CI)	*P*-value	n	Weighted % (95% CI)	*P*-value
Adults ≥18 years	9066	373	3.9 (3.3-4.6)	–	178	2.0 (1.6-2.6)	–
**Age, years**							
18-29	1898	27	2.4 (1.6-3.8)	<0.001	16	1.6 (0.8-3.3)	0.01
30-39	2358	61	2.5 (1.7-3.7)		26	1.2 (0.6-2.1)	
40-49	1775	70	3.8 (2.5-5.8)		36	2.0 (1.2-3.4)	
50-59	1542	95	6.0 (4.6-7.8)		37	2.4 (1.6-3.5)	
≥60	1493	120	7.7 (6.0-9.7)		63	3.9 (2.9-5.4)	
**Sex**							
Male	3245	119	3.6 (2.6-4.9)	0.46	64	1.9 (1.2-3.0)	0.79
Female	5821	254	4.2 (3.4-5.3)		114	2.1 (1.5-2.9)	
**Region**							
Andijan	1555	64	3.4 (2.7-4.2)	<0.001	32	1.3 (0.6-2.7)	<0.001
Kashkadarya	1463	34	2.2 (1.3-3.6)		20	1.3 (0.7-2.5)	
Khorezm	844	81	8.8 (6.7-11.4)		37	4.6 (2.4-8.6)	
Karakalpakstan	911	40	3.3 (2.3-4.7)		19	1.7 (1.1-2.5)	
Samarkand	1546	56	3.2 (2.1-4.8)		25	1.8 (1.0-3.2)	
Tashkent city	1279	67	6.6 (4.5-9.5)		29	3.1 (1.9-4.9)	
Tashkent region	1468	31	1.8 (1.1-3.1)		16	1.2 (0.7-1.9)	
**Ethnicity**							
Uzbek	7091	278	3.6 (2.9-4.5)	0.28	132	1.9 (1.4-2.6)	0.70
Other	1231	55	4.4 (3.3-5.8)		28	2.2 (1.4-3.4)	
**Marital status**							
Never married	866	16	1.2 (0.6-2.5)	0.001	13	1.2 (0.6-2.4)	0.25
Married/living with partner	6503	272	4.2 (3.4-5.1)		129	2.1 (1.5-2.9)	
Separated/divorced/widowed	701	41	5.3 (3.4-8.1)		15	1.7 (1.0-3.2)	
**Highest level of education completed**							
Secondary school or less	218	6	2.4 (0.6-9.4)	0.75	3	2.0 (0.4-10.0)	0.36
Professional/technical school	3320	143	4.0 (3.0-5.2)		71	2.3 (1.6-3.5)	
University/college or higher	4306	167	3.8 (3.1-4.7)		75	1.6 (1.2-2.2)	
**Employment status**							
Employed	3289	123	4.3 (3.1-5.7)	<0.001	48	2.0 (1.3-3.1)	0.11
Retired	1345	100	7.0 (5.2-9.2)		50	3.2 (2.3-4.5)	
Homemaker	2145	70	3.0 (2.2-4.1)		32	1.3 (0.9-2.0)	
Unemployed/student	1275	34	2.2 (1.2-3.9)		21	1.7 (0.9-3.3)	
**Monthly household income**^a^							
<2,000,000 Som (<178 USD)	2784	136	4.4 (3.4-5.6)	0.60	64	2.4 (1.6-3.7)	0.50
2,000,000-4,000,000 Som (178-356 USD)	2002	73	3.8 (2.6-5.5)		32	1.8 (1.1-2.8)	
>4,000,000 Som (>356 USD)	799	28	3.3 (2.0-5.6)		15	1.8 (0.9-3.6)	
Unknown	1603	56	3.4 (2.4-4.9)		33	1.9 (1.3-2.7)	
**Invasive dental procedure, ever**							
Yes	3444	130	4.5 (3.4-5.9)	0.008	63	2.5 (1.8-3.4)	0.03
No	1508	45	2.4 (1.7-3.3)		20	1.0 (0.5-1.7)	
**Blood transfusion, ever**							
Yes	404	32	6.8 (4.2-10.8)	0.03	14	3.0 (1.6-5.3)	0.26
No	7687	303	3.9 (3.2-4.6)		143	2.0 (1.5-2.6)	
**Surgery or invasive medical procedure, ever**							
Yes	2248	135	6.4 (4.9-8.2)	<0.001	63	3.1 (2.4-4.1)	0.004
No	6038	202	3.0 (2.5-3.8)		98	1.6 (1.1-2.3)	
**Injection drug use, ever**							
Yes	37	2	1.9 (0.2-15.4)	0.48	1	0.3 (0.0-3.8)	0.10
No	7324	294	4.0 (3.3-4.8)		137	2.0 (1.5-2.7)	
**History of incarceration**							
Yes	66	9	14.5 (4.6-37.1)	0.01	2	4.4 (0.7-24.1)	0.37
No	7447	290	3.8 (3.2-4.4)		141	2.0 (1.5-2.6)	
**Receipt of a tattoo**							
Yes	182	20	11.9 (5.7-23.0)	<0.001	12	7.4 (3.4-15.2)	<0.001
No	6892	262	3.7 (3.1-4.4)		126	1.9 (1.4-2.6)	
**Piercings**							
Yes	1198	60	4.0 (2.9-5.5)	0.96	27	1.7 (1.1-2.7)	0.23
No	4721	171	4.0 (3.2-5.1)		84	2.3 (1.7-3.2)	
**Share household items**							
Yes	1411	42	3.5 (2.8-4.4)	0.42	22	2.2 (1.4-3.5)	0.78
No	5155	217	4.0 (3.1-5.2)		102	2.0 (1.4-2.9)	

Abbreviations: CI, confidence interval; HCV, hepatitis C virus; USD, US dollars.

a Conversion rate to USD as of May 1, 2022 from https://www.oanda.com/currency-converter

**Table 3:** Weighted prevalence (% and 95% CI) of hepatitis C among children by socio-demographic characteristics, Uzbekistan, 2022.

Characteristics	Total	Anti-HCV+			HCV RNA+		
	N	n	Weighted % (95% CI)	*P*-value	n	Weighted % (95% CI)	*P*-value
**Overall**	4087	44	1.1 (0.5-2.2)	–	19	0.5 (0.2-1.0)	–
**Age, years**							
5-9	1726	12	0.8 (0.2-3.2)	0.08	2	0.1 (0.0-0.6)	<0.001
10-14	1599	11	0.8 (0.4-1.8)		6	0.3 (0.1-0.7)	
15-17	762	21	2.4 (1.2-4.7)		11	1.8 (0.7-4.3)	
**Sex**							
Male	2001	19	0.7 (0.4-1.3)	0.15	8	0.4 (0.1-0.9)	0.54
Female	2086	25	1.5 (0.6-4.0)		11	0.6 (0.2-1.9)	
**Region**							
Andijan	659	19	1.1 (0.3-3.8)	<0.001	7	0.5 (0.1-2.1)	–
Kashkadarya	580	13	3.2 (0.9-10.8)		7	1.0 (0.2-4.0)	
Khorezm	394	5	2.5 (0.9-6.8)		4	2.0 (0.6-6.1)	
Karakalpakstan	478	0	–		0	–	
Samarkand	689	5	0.5 (0.2-1.4)		0	–	
Tashkent city	558	1	0.1 (0.0-0.7)		0	–	
Tashkent region	729	1	0.2 (0.0-1.4)		1	0.2 (0.0-1.4)	
**Ethnicity**							
Uzbek	2853	30	0.6 (0.3-1.2)	0.33	11	0.3 (0.1-0.7)	0.38
Other	415	1	0.2 (0.0-1.9)		0	–	

Abbreviations: CI, confidence interval; HCV, hepatitis C virus.

### *Prevalence of HCV RNA*

Overall, among persons aged ≥5 years, the prevalence of HCV RNA was 1.6% (95% CI: 1.3-2.0). The prevalence of HCV RNA among adults was 2.0% (95% CI: 1.6-2.6). Prevalence of HCV RNA was lowest among those aged 30-39 years (1.2% [95% CI: 0.6-2.1]) and then increased with age to 3.9% (95% CI: 2.9-5.4) among those aged ≥60 years (*P* < 0.001). Prevalence was similar for males (1.9% [95% CI: 1.2-3.0]) and females (2.1% [95% CI: 1.5-2.9]) but varied by region, with the highest prevalence (4.6% [95% CI: 2.4-8.6] among residents of Khorezm. HCV RNA prevalence did not significantly differ by ethnicity, marital status, education, employment, or injection drug use. Prevalence was higher among those who reported undergoing invasive dental procedures, surgery or invasive medical procedures, or having a tattoo (Table 2). Overall, among adults positive for HCV RNA, 12.1% (95% CI: 1.4-22.7) reported that they had been previously informed by a health care provider that they had hepatitis C. Among children, the overall HCV RNA prevalence was 0.5% (95% CI: 0.2-1.0), which increased with age from 0.1% (95% CI: 0.0-0.6) among those aged 5-9 years to 1.8% (95% CI: 0.7-4.3) among those aged 15-17 years (Table 3). HCV RNA prevalence was 0.4% (95% CI: 0.1-0.9) among male children and 0.6% (95% CI: 0.2-1.9) among female children (*P* = 0.54).

### *Factors associated with anti-HCV positivity*

In bivariate analysis, among adults, prevalence of anti-HCV was associated with age (*P* < 0.001), region (*P* < 0.001), marital status (*P* = 0.001), employment status (*P* < 0.001), receipt of a blood transfusion (*P* = 0.03), ever having undergone an invasive dental procedure (*P* = 0.008), ever having undergone surgery or an invasive medical procedure (*P* < 0.001), receipt of a tattoo (*P* < 0.001), and a history of incarceration (*P* = 0.01) (Table 2). After adjusting for all covariates associated with anti-HCV positivity, ever having undergone surgery or an invasive medical procedure (aOR 1.65 [95% CI: 1.09-2.50]), and a history of incarceration (aOR 4.88 [95% CI: 1.46-16.29]) were independently associated with anti-HCV positivity (Table 4). There were also regional differences in prevalence; compared with participants residing in the capital, Tashkent city, those residing in the surrounding Tashkent region (aOR 0.41 [95% CI: 0.22-0.74]) were less likely to be anti-HCV positive, whereas residents of Khorezm (aOR 3.53 [95% CI: 1.93-6.47]) were more likely to be anti-HCV positive.

**Table 4:** Socio-demographic characteristics and exposures associated with hepatitis C prevalence among adults, Uzbekistan, 2022.

Characteristics	Anti-HCV	HCV RNA
	Adjusted^a^ OR (95% CI)	Adjusted^b^ OR (95% CI)
**Age, years**		
18-29	1	1
30-39	0.53 (0.17-1.62)	0.47 (0.12-1.79)
40-49	0.66 (0.23-1.88)	0.95 (0.26-3.50)
50-59	1.31 (0.46-3.74)	1.17 (0.36-3.83)
≥60	1.30 (0.37-4.58)	1.61 (0.48-5.35)
**Sex**		
Male	1	1
Female	0.82 (0.43-1.58)	0.92 (0.38-2.21)
**Region**		
Tashkent city	1	1
Andijan	1.30 (0.70-2.42)	1.30 (0.40-4.28)
Kashkadarya	0.50 (0.19-1.33)	0.88 (0.28-2.73)
Khorezm	3.53 (1.93-6.47)	3.13 (0.85-11.54)
Karakalpakstan	0.85 (0.48-1.51)	0.93 (0.47-1.82)
Samarkand	0.81 (0.42-1.56)	1.53 (0.70-3.37)
Tashkent region	0.41 (0.22-0.74)	0.61 (0.30-1.25)
**Invasive dental procedure, ever**		
Yes	1.66 (0.77-3.61)	2.63 (1.11-6.22)
No	1	1
**Blood transfusion, ever**		
Yes	1.62 (0.79-3.31)	
No	1	
**Surgery or invasive medical procedure, ever**		
Yes	1.65 (1.09-2.50)	1.73 (0.98-3.07)
No	1	1
**History of incarceration**		
Yes	4.88 (1.46-16.29)	
No	1	
**Receipt of a tattoo**		
Yes	1.87 (0.91-3.84)	3.84 (1.57-9.38)
No	1	1

Abbreviations: CI, confidence interval; HCV, hepatitis C virus; OR, odds ratio.

a Adjusted for marital status and employment status in addition to variables shown.

b Adjusted for all variables with results shown.

### *Factors associated with HCV RNA positivity*

In bivariate analysis, among adults, the prevalence of HCV RNA was associated with age (*P* = 0.01), region (*P* < 0.001), ever having undergone an invasive dental procedure (*P* = 0.03), ever having undergone surgery or an invasive medical procedure (*P* = 0.004), and receipt of a tattoo (*P* < 0.001) (Table 2). After adjusting for all covariates associated with HCV RNA positivity, having undergone an invasive dental procedure (aOR 2.63 [95% CI: 1.11-6.22]) and receipt of a tattoo remained independently associated with HCV infection (aOR 3.84 [95% CI: 1.57-9.38]) (Table 4). Among persons who reported having a tattoo, 43.1% (95% CI: 25.2-63.1) said they were tattooed in a beauty or tattoo salon, 16.8% (95% CI: 8.3-31.2) in the army, 12.0% (95% CI: 5.9-22.8) at home, and 9.5% (95% CI: 3.7-22.1) in prison settings. Twelve participants with HCV infection reported having a tattoo. While four reported getting a tattoo in a tattoo parlor, the remaining persons (n = 8) received their tattoos in non-professional settings (prison, army, or home).

## Discussion

This analysis presents the most robust prevalence estimates for hepatitis C in the selected regions of Uzbekistan. In the seven regions selected, which accounted for nearly 60% of Uzbekistan’s population, the prevalence of anti-HCV and HCV RNA was 3.9% and 2.0%, respectively, among adults and 1.1% and 0.5%, respectively, among children in 2022. This prevalence is considerably lower than that reported in a 2019 meta-analysis that estimated the prevalence of anti-HCV to be 9.6% (95% CI: 5.8-14.2) [10], but higher than modeled estimates suggesting that 1.1% of persons in Uzbekistan were living with HCV infection in 2022 [11]. We also found an anti-HCV prevalence of 6.6% in Tashkent city, which is considerably higher than the 3.0% observed in the 2019 polyclinic-based pilot program conducted in the city [7].

The prevalence of HCV RNA in Uzbekistan is lower than that reported in several countries in Eastern Europe and Central Asia, where prevalence can be 3-5% [12] but is similar to the prevalence among adults in the nearby country of Georgia (1.8%) in 2021, which initiated a hepatitis C elimination program in 2015 [13]. Among the 18.1 million persons aged ≥5 years in these seven regions in 2022 [4], an HCV RNA prevalence of 1.6% (95% CI: 1.3-2.0) in the general population equates to 289,500 (95% CI: 235,000-362,000) persons with chronic HCV infection (HCV RNA positive). Further extrapolation to Uzbekistan’s national population of 31.3 million aged ≥5 years would equate to 501,000 (95% CI: 407,000-627,000) persons with hepatitis C, which is a considerable burden. Hepatitis C elimination programs have been shown to be effective at significantly reducing prevalence [13] but require a multi-pronged approach to meet elimination targets, which include reducing incidence and mortality [14]. Uzbekistan has already taken the first steps by officially launching a national viral hepatitis elimination program and offering screening, diagnostic testing, and treatment free of charge. Additionally, an initiative conducted during 2022-2025, subsequent to this survey, aimed to screen 500,000 persons per year for hepatitis B and hepatitis C, and in its first two years identified approximately 20,000 persons with current HCV infection and linked them to care [15]. As efforts progress, the findings of this study can help guide Uzbekistan in its journey toward hepatitis C elimination, both as a baseline assessment and as epidemiologic evidence of areas requiring focus.

Prevalence of both anti-HCV and HCV RNA increased significantly with age in Uzbekistan, and ≥2% of persons aged ≥40 years had chronic HCV infection. The trend of prevalence increasing with age was also observed in the 2019 pilot program in Tashkent [7]. These results provide valuable insight for developing targeted, age-based hepatitis C testing initiatives to identify persons with HCV infection and link them to care and treatment. In the United States, for example, birth-cohort testing was initially recommended for persons born during 1945-1965 after recognizing their disproportionately high prevalence of hepatitis C [16], and later expanded to all adults aged ≥18 years [17]. Such initiatives in the United States and other countries have been shown to be a cost-effective approach for identifying persons in need of treatment [18,19], and could be considered in Uzbekistan by starting to screen those aged ≥40 years.

The higher prevalence of hepatitis C among older age groups could be indicative of iatrogenic infections due to greater cumulative exposure to health care services and poor infection prevention and control practices and blood safety in the past [20]. Health care-associated transmission of viral hepatitis is well documented [21], and our analysis found that past or present HCV infection (i.e. anti-HCV positivity) among adults was associated with ever having undergone surgery or an invasive medical procedure, and that chronic HCV infection was associated with ever having undergone an invasive dental procedure. This suggests that a focus on infection prevention and control procedures could mitigate the burden of hepatitis C in Uzbekistan. Awareness campaigns among health care workers, including dental workers, could be considered to not only promote adherence to necessary infection prevention and control protocols but also to increase awareness and promote testing and treatment uptake among the general population to facilitate elimination goals [9]. This may particularly be considered in more rural areas, such as the region of Khorezm, where residents had more than three times the odds of having past or present HCV infection compared with residents of the capital, Tashkent.

History of incarceration was also associated with anti-HCV positivity. Ensuring that persons in carceral settings have access to hepatitis C testing and treatment is important for preventing new infections. Hepatitis C treatment as a means of prevention has previously been demonstrated in prison settings and is a vital consideration for HCV elimination strategies [22]. The resultant prevalence reduction in carceral settings could also help prevent transmission in the general population after those cured of their infection are eventually released. Targeted screening campaigns among persons who are incarcerated should also be considered to better characterize the epidemic and increase awareness of infection status among these populations.

We also found that receipt of a tattoo was independently associated with chronic HCV infection. Previous studies have shown that there is a risk of HCV infection from tattoos received in non-professional settings [23]. Our findings indicate that only approximately half of adults with tattoos received them in a professional salon, with the others receiving their tattoos while in the army, at home, or in carceral settings. The majority of persons with HCV infection received their tattoos in non-professional settings. The low number of participants in our study who were HCV RNA positive prevented further analysis of infection by tattoo venue, but future assessments could be considered to elucidate these potential risks. Educating the public on the importance of receiving tattoos under sterile conditions and implementing training on infection prevention and control for tattoo parlors could also help to prevent HCV transmission in these circumstances.

The low hepatitis C prevalence in children and adolescents in Uzbekistan could be due to the substantially lower levels of behaviors associated with non-health care-related transmission (e.g., sexual contacts, injection drug use, non-medical invasive procedures, and incarceration) compared with adults. Improvement in health care safety standards over time could also have contributed to the lower prevalence among children. The increase in prevalence among those aged 15-17 years compared with younger age groups needs to be further investigated to determine the risk factors in this age group.

This study is subject to several limitations. The cross-sectional nature of the survey limits interpretation of factors associated with past or present infection. Since a person could have been infected at any time during their life, practices and exposures associated with increased risk for infection could have taken place after exposure to HCV and thus are not necessarily the cause of infection. Self-reported behaviors, such as medical exposures, may have been subject to recall bias, and others, such as injection drug use, may also have been underreported due to social desirability bias and the criminalization of drug use [24]. The relatively low number of persons testing HCV RNA positive also likely affected risk factor analysis, particularly for infrequently reported potential exposures like injection drug use or a history of incarceration, which could explain differences in associated factors for anti-HCV and HCV RNA. We were also unable to assess potential risk factors among adolescents due to cultural sensitivities. Although a majority of Uzbekistan’s population resided in the regions selected for the survey, half of the country’s regions were not sampled, which limits generalizability to the overall population, and thus the true national prevalence of hepatitis C may differ from our findings. Nearly two-thirds of participants in our study were female. Although post-stratification weighting was conducted to adjust for this, our sample may not have been representative of the general population. Lastly, there was a substantial proportion of missing values for some variables, which limited the analytic power for some findings.

Uzbekistan has been working toward the elimination of hepatitis C as a public health threat since 2017 and offers free testing and treatment to the general population. To bolster elimination efforts by identifying persons with HCV infection and linking them to care, Uzbekistan could consider implementing nationwide screening that focuses on adults aged ≥40 years and persons with a history of incarceration. Implementation of infection prevention and control measures in health care settings should also be considered to help prevent future infections.

## CRediT authorship contribution statement

**Shaun Shadaker:** Formal analysis, Methodology, Visualization, Writing – original draft, Writing – review & editing. **Elizaveta Joldasova:** Project administration, Writing – review & editing. **Rania A. Tohme:** Methodology, Project administration, Writing – review & editing. **Aybek Khodiev:** Project administration, Writing – review & editing. **Shakhida Karamatova:** Project administration, Writing – review & editing. **Nataliya Kan:** Project administration, Writing – review & editing. **Abdulaziz Abdurasulov:** Project administration, Writing – review & editing. **Botirjon Kurbanov:** Project administration, Writing – review & editing. **Zulfiya Abdurakhimova:** Project administration, Writing – review & editing. **Dilafkor Mirdjalilov:** Project administration, Writing – review & editing. **Saleem Kamili:** Methodology, Project administration, Writing – review & editing. **Nino Khetsuriani:** Methodology, Project administration, Writing – review & editing. **Erkin Musabaev:** Conceptualization, Supervision, Writing – review & editing.

## Declaration of competing interest

The authors declare that they have no known competing financial interests or personal relationships that could have appeared to influence the work reported in this paper.
